# Comparison of ropivacaine combined with sufentanil for epidural anesthesia and spinal-epidural anesthesia in labor analgesia

**DOI:** 10.1186/s12871-019-0855-y

**Published:** 2020-01-02

**Authors:** Yanshuang Wang, Mingjun Xu

**Affiliations:** Department of Anesthesiology, Beijing Obstetrics and Gynecology Hospital, Capital Medical University, Beijing, 100029 China

**Keywords:** Ropivacaine, Sufentanil, Continuous epidural anesthesia, Combined spinal-epidural anesthesia, Labor analgesia

## Abstract

**Background:**

To compare the application and efficacy of ropivacaine combined with sufentanil for continuous epidural anesthesia (CEA) and combined spinal-epidural anesthesia (CSEA) in labor analgesia.

**Methods:**

Three hundred sixty pregnant women requesting labor analgesia from October 2017 to August 2018 were selected retrospectively. According to the anesthetic method, subjects were divided into CSEA group and CEA group. Ropivacaine combined with sufentanil were used in all subjects. The labor time, visual analogue scale (VAS), Apgar score of newborn, adverse pregnancy outcomes and adverse drug reactions were observed.

**Results:**

There was no significant difference in pre-analgesia (T_0_) VAS scores between the two groups (*P* > 0.05). VAS scores of first stage of labor (T_1_), second stage of labor (T_2_) and third stage of labor (T_3_) in CSEA group were significantly lower than CEA group (*P* < 0.01). The onset time, T_1_ and total labor time in CSEA group were significantly shorter than CEA group (*P* < 0.01). There were no significant differences between T2 and T3 (*P* > 0.05). There were no significant differences in adverse pregnancy outcomes and Apgar scores at 1, 5 and 10 min after birth between the two groups (*P* > 0.05). The incidence of adverse drug outcomes in CSEA group was significantly lower than CEA group (*P* < 0.01). Maternal satisfaction in CSEA group was significantly higher than CEA group (*P* < 0.01).

**Conclusion:**

Considering ropivacaine combined with sufentanil for CSEA achieved a shorter onset time and labor period, significant analgesic effect, lower adverse drug reactions rates and higher subject satisfaction than CEA, it may be worthy of clinical promotion and application.

## Background

Childbirth is usually the most challenging and painful experience of a mother’s life [1]. Fear of childbirth has increased the cesarean delivery rate. Due to the fact that natural vaginal delivery has many advantages compared with cesarean section and promotion of natural vaginal delivery is considered important by the health care systems, labor analgesia has become a key concern for both puerperal and clinical anesthesiologists [2].

At present, labor analgesia methods include non- pharmacological analgesia and pharmacological analgesia. Non-pharmacological analgesia mainly includes acupuncture, massage, yoga and hydrotherapy and the analgesic effect of which is poor although there is no maternal and fetal injury [3]. For pharmacological analgesia, there are intramuscular analgesics, intravenous analgesics, and spinal analgesia. Among them, intraspinal labor analgesia is the best method for clinical application of analgesia, including continuous epidural analgesia (CEA), combined spinal-epidural anesthesia (CSEA) and continuous spinal anesthesia [4, 5]. Epidural analgesia is considered as a widely practiced analgesic technique in clinic worldwide [6]. An ideal labor analgesia method should exhibit good analgesic effect, increase subject satisfaction and comfort, and reduce the incidence of adverse pregnancy outcomes and adverse drug reactions without affecting the progress of labor [7].

In recent years, with the development of anesthesia technology, combined spinal-epidural anesthesia has increasingly used in labor analgesia because it combines the advantages of epidural anesthesia and spinal anesthesia, including rapid onset, significant analgesic effect, lower drug dosage and fewer side effects [8, 9]. However, there are few reports on the specific anesthesia methods and medication regimens of combined spinal-epidural anesthesia, which is not conducive to guiding clinical work. Here, we compared the clinical effects of combined spinal-epidural anesthesia and continuous epidural anesthesia, to provide guidance for clinical work.

## Methods

### Study design and population

In this retrospective, single-center case-control analysis, a total of 360 cases of voluntary acceptance of painless childbirth pregnant from October 2017 to August 2018 in the Beijing Obstetrics and Gynecology Hospital were selected in this study retrospectively. The inclusion criteria were: (1) primiparous women; (2) at first full-term pregnancy, singleton pregnancies; and (3) cephalic, and had no spinal deformity. Exclusion criteria were: (1) pregnant women who had contraindication to epidural anesthesia; (2) pregnant women who had signs of fetal distress; (3) pregant women with contraindications for vaginal delivery. The subjects were divided into observation group and control group according to anesthetic method (*n* = 180 in each group, control: case were 1:1). For the observation group, combined spinal-epidural anesthesia (CSEA group) was used. Cases in control group received continuous epidural anesthesia (CEA group). Ropivacaine combined with sufentanil were used in all subjects. All subjects were American Society of Anesthesiologists (ASA) physical status class I or II. One hundred forty-two subjects in CSEA group and 133 subjects in CEA group were class I and 18 subjects in CSEA group and 27 subjects in CEA group were class II.

### Anesthetic method

After entering the clinical delivery, the routine monitoring was established for all women, including blood pressure (BP), heart rate (HR), pulse oximetry (SpO2) and electrocardiography (ECG). Oxygen therapy by nasal catheter venous access and fetal heart monitoring was given. In terms of labor analgesia, the impact on the patient’s circulation and other aspects is very small since the dosage of CSEA injected into the subarachnoid space is only 1/5–1/10 of normal CSEA anesthesia. In addition, CEA and CSEA were performed in patients with normal coagulation function. Therefore, there is no obvious tendency in the selection of indications. Both CEA and CSEA were routinely used in our department and the method was chosen according to the anesthesiologist’s personal anesthesia operation habits.

For CEA group, pregnant women were placed in the left lateral position. After successful epidural puncture at L2–3 or L3–4 interspace, 3 mL of 0.5% ropivacaine (Naropin; AstraZeneca Co., Ltd.; 10 ml/100 mg; Registration number: H20140763) were slowly injected. If no occurrence of adverse reactions were observed 10 min later, pre-configured 10 mL of analgesic pump solution were given from the epidural space and epidural analgesia analgesia pump (PCEA) was then connected. The formula of analgesic pump solution was: 10 ml of l% ropivacaine combined with 0.3–0.4 μg/ml sufentanil (Eurocept BV Trapgans 51,244 RL Ankeveen, The Netherlands; 75 μg/1 ml; Batch number: 170553) diluted to 100 ml with 0.9% sterile saline. The analgesic pump parameters were set as follows: continuous background infusion of 5 ml/h, single additional dose (PCA) of 7 ml/time, interval lockout time of 15 min and total amount of 100 ml.

For pregnant women in CSEA group, after successful epidural puncture at L2–3 or L3–4 interspace, a 25-G lumbar puncture needle was inserted through the epidural needle (Xinxiang Camel Medical Devices Co., Ltd.; Batch number: 1310015). After the cerebrospinal fluid was seen 2–3 mg of 0.1% ropivacaine (diluted to 2–3 ml with sterile saline) was then injected into the subarachnoid space. After 3–4 cm of epidural catheter was set to the head side, subjects in both groups were placed in the supine position. Ten minutes later, epidural analgesia analgesia pump (PCEA) was connected after no occurrence of adverse reactions were observed. The formula of analgesic pump solution and analgesic pump parameters were the same as CEA group.

For both groups, the heart rate, blood pressure and fetal heart rate of the pregnant women were closely monitored during the process of analgesia and childbirth. After the uterine cervix was opened, the pump fluid infusion was stopped, and the epidural catheter was pulled out 2 h after delivery.

### Observation indexes

The onset time of analgesia, T1, T2, T3, total labor time and doses of ropivacaine and sufentanil in two groups were recorded. Pain was assessed by visual analog scale (VAS) [10] scores at four time points: (1) T_0_, stage before analgesia; (2) T_1_, from the beginning of uterine contractions to cervical dilatation of 10 cm; (3) T_2,_ from cervical dilatation of 10 cm to the delivery of the baby; (4) T_3_, From the delivery of the baby to the delivery of the placenta. The T_0_, T_1_, T_2_ and T_3_ in this study were defined according to the guidelines from the National Institute for Health and Clinical Excellence [11].

The Apgar scores were assessed at 1, 5, and 10 min after birth [12]. Apgar scores of 8–10 points were considered normal for newborns; 4–7 points indicated mild asphyxia; and 0–3 points suggested severe asphyxia.

Maternal satisfaction evaluation were performed based on subjects’ feelings during different labor periods on labor analgesia (80–100: very satisfactory; 60–80: general satisfactory; below 60: unsatisfactory).

Finally, the adverse pregnancy outcomes and adverse drug reactions were observed.

### Statistical analysis

All statistical analyses were conducted with SPSS software, version 18.0 (SPSS Inc., Chicago, USA). PP plot (probability-probability plot) indicates the normal distribution of data. Values are presented as mean ± standard deviation (SD) or number (percentage) at appropriate. Quantitative variables (VAS scores, labor time, apgar scores and doses of ropivacaine and sufentanil) were compared by *Student’s t test* and χ2 test was used for categorical variables (adverse pregnancy outcomes and adverse drug reactions). Repeated-measures ANOVA were used for the comparison of different time points within the group. All reported *P* values are two-sided, and *P* value of less than 0.05 was considered statistically significant.

## Results

There were no significant differences with regard to age, height, weight, BMI and duration of pregnancy between CSEA group and CEA group (*P* > 0.05) (Table 1).

**Table 1:** Clinical characteristics of subjects (mean ± SD)

	CEA (*n* = 180)	CSEA (*n* = 180)	*P* value
Age, years	26.8 ± 4.2	27.1 ± 3.9	0.483
Height, cm	160.4 ± 4.3	161.3 ± 4.6	0.056
Weight, kg	65.4 ± 16.2	66.3 ± 15.6	0.592
BMI, kg/m^2^	22.3 ± 3.4	22.2 ± 3.1	0.771
Duration of pregnancy, weeks	38.5 ± 1.3	38.3 ± 1.4	0.161

*BMI* Body Mass Index. *P* value of comparison between CSEA and CEA groups

### VAS scores in two groups

There was no significant differenceregarding VAS scores at T_0_ between two groups (*P* > 0.05). In two groups, VAS scores at T_1_, T_2_ and T_3_ were significantly lowercompared with T_0_ in the same group (*P* < 0.01). VAS scores at T_1_, T_2_ and T_3_ in CSEA group were significantly lower compared with those in CEA group at the same time (*P* < 0.01) (Table 2).

**Table 2:** Comparison of pain VAS scores at different times in two groups (mean ± SD)

	CEA (*n* = 180)	CSEA (*n* = 180)
T_0_ (min)	8.32 ± 1.37	8.36 ± 1.35
T_1_(min)	3.35 ± 0.64^*^	3.02 ± 0.67^*#^
T_2_ (min)	4.22 ± 0.87^*^	3.84 ± 0.83^*#^
T_3_ (min)	6.84 ± 1.26^*^	5.31 ± 1.12^*#^

^*^P < 0.01, vs T_0_; ^#^*P* < 0.01, vs CEA group

### Labor time in two groups

The onset time, T_1_ and total labor time in CSEA group were significantly shorter than those in CEA group (*P* < 0.01). However, there was no significant difference regarding labor time at T_2_ and T_3_ between two groups (*P* > 0.05) (Table 3).

**Table 3:** Comparison of labor time in two groups (mean ± SD)

	CEA (*n* = 180)	CSEA (*n* = 180)	*P* value
Onset time (min)	6.32 ± 3.45	3.21 ± 1.42	< 0.001
T_1_(min)	524.33 ± 126.46	468.32 ± 114.10	< 0.001
T_2_(min)	48.24 ± 10.34	46.09 ± 11.22	0.060
T_3_(min)	7.38 ± 0.76	7.24 ± 0.83	0.096
Total labor time (min)	578.29 ± 132.08	512.67 ± 124.38	< 0.001

### Apgar scores in two groups

There were no significant differences in Apgar scores at 1, 5, and 10 min after birth between the two groups (*P* > 0.05) (Table 4).

**Table 4:** The Apgar scores at 1, 5, and 10 min after birth in two groups (mean ± SD)

	CEA (*n* = 180)	CSEA (*n* = 180)	*P* value
1 min after birth	8.47 ± 0.34	8.41 ± 0.35	0.099
5 min after birth	8.85 ± 0.41	8.83 ± 0.42	0.647
10 min after birth	9.52 ± 0.38	9.48 ± 0.33	0.278

### Adverse pregnancy outcomes in two groups

There were no significant differences in adverse pregnancy outcomes after labor analgesia between the two groups (*P* > 0.05) (Table 5), including obstetric apparatus, cesarean, 24 h postpartum hemorrhage and episiotomy.

**Table 5:** Comparison of adverse pregnancy outcomes in two groups (mean ± SD)

	CEA (*n* = 180)	CSEA (*n* = 180)	*P* value
Obstetric apparatus, n (%)	7 (3.89)	8 (4.45)	0.792
Cesarean, n (%)	18 (10.00)	22 (12.22)	0.499
24 h postpartum hemorrhage, n (%)	6 (3.33)	5 (2.78)	0.759
Episiotomy, n (%)	22 (12.22)	16 (8.89)	0.303
Total adverse pregnancy outcomes, n (%)	44 (24.44)	41 (22.78)	0.709

### Doses of drugs and adverse drug reactions in two groups

Doses of ropivacaine and sufentanil in CSEA group were significantly lower than that in CEA group (*P* < 0.01) (Table 6). There were no significant differences in the rates of uroschesis and respiratory depression between two groups (*P* > 0.05). However, the rates of nausea, vomiting, pruritus and total adverse drug reactions in CSEA group were significantly lower than these in CEA group (*P* < 0.01). Besides, subject satisfaction in CSEA group were significantly higher than that in CEA group (*P* < 0.01) (Table 7).

**Table 6:** Doses of Ropivacaine and Sufentanil in each group. (mean ± SD)

	CEA (*n* = 180)	CSEA (*n* = 180)	*P* value
Ropivacaine	39.88 ± 4.52	38.42 ± 3.16	< 0.001
Sufentanil	19.98 ± 6.66	16.01 ± 5.26	< 0.001

**Table 7:** Comparison of adverse drug reactions in two groups

	CEA (*n* = 180)	CSEA (*n* = 180)	*P* value
Nausea and vomiting, n (%)	20 (11.11)	8 (4.44)	0.018
Pruritus, n (%)	12 (6.67)	4 (2.22)	0.041
Uroschesis, n (%)	8 (4.44)	4 (2.22)	0.240
Respiratory depression, n (%)	4 (2.22)	2 (1.11)	0.410
Total adverse drug reactions, n (%)	44 (24.44)	18 (10.00)	< 0.001
Subject satisfaction, n (%)	120 (66.67)	150 (83.33)	< 0.001

## Discussion

Under the different combinations of ropivacaine and sufentanil, the observation and analysis of the two anesthesia methods in this study showed that CSEA was superior to CEA in analgesic effect and maternal satisfaction.

CSEA refers to a method of injecting a small amount of anesthetic drug into the subarachnoid space after successful epidural puncture [13]. Currently, the combination of local anesthetic and opioid is the common compatibility program for CSEA, including bupivacaine, ropivacaine, fentanyl and sufentanil [14, 15]. Ropivacaine (1-propyl-2′, 6′-pipecoloxylidide, chemical structure) is an amino acid local anesthetic and was recently introduced for labor analgesia, since it has less cardiac toxicity and provide more differential block between sensory and motor [7, 16]. For opioids, the analgesic effect of sufentanil is 3 to 5 times more potent than fentanyl due to the strong affinity for opioid receptors [17, 18].

Clinically, ropivacaine combined with low-dose sufentanil has been widely used in labor analgesia [19]. The combination of the two drugs allows use of lower doses of both drugs, thus decreasing the rate and severity of adverse effects [20]. However, previous studies [21, 22] have shown that ropivacaine combined with sufentanil injected into the subarachnoid space after the spinal anesthesia pierced the dura mater resulted in a higher pruritus rates than ropivacaine alone. In this study, ropivacaine combined with sufentanil were used for CEA group. While for CSEA group, ropivacaine were used alone before epidural catheter were placed and ropivacaine combined with sufentanil were used thereafter. As a result, better analgesic effect was achieved in CSEA group compared with CEA group according to the VAS scores. Besides, the overall incidence of adverse drug reactions in CSEA group was lower than CEA group, including the lower nausea and vomiting rates and pruritus rates. The reduced dose of sufentanil were responsible for this result since complications of anesthetics were dose-dependent [18, 23]. Additionally, delaying sufentanil injection may exert the maximum effect of ropivacaine, which has long analgesic duration, less adverse drug reactions and differential block between sensory and motor [24].

In this study, CSEA group has a relative shorter onset time compared with CEA group, which is inconsistent with a previous study [25], showing that there were no differences between groups for onset time and analgesia evaluation. We assume that this possibly due to the different doses of ropivacaine and sufentanil in both studies. We found no statistically significant differences with regard to the Apgar scores of the newborns at 1, 5 and 10 min and adverse pregnancy outcomes between the two groups suggesting that CSEA could achieve the same newborn conditions as CEA with lower doses of ropivacaine and sufentanil. Maternal satisfaction were improved due to the fewer adverse drug reactions associated with intrathecal sufentanil that can be disconcerting for subjects.

The limitation of this study was that pregnant women with contraindication to epidural anesthesia were not suitable for this method. Therefore, more studies should be carried out in the future to further improve the labor analgesia method.

## Conclusion

CSEA group in this study achieved better analgesic effect, lower onset time and fewer adverse drug reactions compared with CEA group. Thus, CSEA method with our block method and doses may provide some reference for clinical labor analgesia.

## Data Availability

The datasets used and/or analysed during the current study available from the corresponding author on reasonable request.

## References

[1] Chajut E, Caspi A, Chen R, Hod M, Ariely D (2014). In pain thou shalt bring forth children: the peak-and-end rule in recall of labor pain. Psychol Sci.

[2] Attar AS, Feizabadi AS, Jarahi L, Feizabadi LS, Sheybani S (2016). Effect of Entonox on reducing the need for Pethidine and the relevant Fetal and maternal complications for painless labor. Electron Physician.

[3] Koyyalamudi V, Sidhu G, Cornett EM, Nguyen V, Labrie-Brown C, Fox CJ, Kaye AD (2016). New labor pain treatment options. Curr Pain Headache Rep.

[4] Simmons SW, Taghizadeh N, Dennis AT, Hughes D, Cyna AM (2012). Combined spinal-epidural versus epidural analgesia in labour. Cochrane Database Syst Rev.

[5] McKenzie CP, Carvalho B, Riley ET (2016). The Wiley spinal catheter-over-needle system for continuous spinal Anesthesia: a case series of 5 Cesarean deliveries complicated by Paresthesias and headaches. Reg Anesth Pain Med.

[6] Bos EME, Hollmann MW, Lirk P (2017). Safety and efficacy of epidural analgesia. Curr Opin Anaesthesiol.

[7] Chen SY, Lin PL, Yang YH, Yang YM, Lee CN, Fan SZ, Chen LK (2014). The effects of different epidural analgesia formulas on labor and mode of delivery in nulliparous women. Taiwan J Obstet Gynecol.

[8] Matsumine R, Sumikura H (2013). Analgesic treatment of vaginal delivery for late termination and intrauterine fetal demise during the second or third trimester of pregnancy. J Anesth.

[9] Feng BB, Wang L, Zhai JJ (2013). Investigation on delivery analgesia effect of combined spinal epidural anesthesia plus doula and safety of mother and baby. Clin Exp Obstet Gynecol.

[10] Patrick DW, Ronald M (1985). Textbook of PAIN.

[11] Bick D (2004). Caesarean section. Clinical guideline. National Collaborating Centre for Women's and Children's health: commissioned by the National Institute for clinical excellence. Worldviews Evid-Based Nurs.

[12] Apgar V (1953). A proposal for a new method of evaluation of the newborn infant. Curr Res Anesth Analg.

[13] Okutomi T, Saito M, Mochizuki J, Kuczkowski KM (2009). Combined spinal-epidural analgesia for labor pain: best timing of epidural infusion following spinal dose. Arch Gynecol Obstet.

[14] Lv BS, Wang W, Wang ZQ, Wang XW, Wang JH, Fang F, Mi WD (2014). Efficacy and safety of local anesthetics bupivacaine, ropivacaine and levobupivacaine in combination with sufentanil in epidural anesthesia for labor and delivery: a meta-analysis. Curr Med Res Opin.

[15] Shah OM, Bhat KM (2017). Comparison of the efficacy and safety of morphine and fentanyl as adjuvants to bupivacaine in providing operative Anesthesia and postoperative analgesia in Subumblical surgeries using combined spinal epidural technique. Anesth Essays Res.

[16] Zhao L, Wang Y, Zhai Y, Wang Z, Liu J, Zhai G (2014). Ropivacaine loaded microemulsion and microemulsion-based gel for transdermal delivery: preparation, optimization, and evaluation. Int J Pharm.

[17] Li B, Wang H, Gao C (2015). Bupivacaine in combination with fentanyl or sufentanil in epidural/intrathecal analgesia for labor: a meta-analysis. J Clin Pharmacol.

[18] Coleman L, Carvalho B, Lipman S, Schmiesing C, Riley E (2009). Accidental intrathecal sufentanil overdose during combined spinal-epidural analgesia for labor. Int J Obstet Anesth.

[19] Bremerich DH, Waibel HJ, Mierdl S, Meininger D, Byhahn C, Zwissler BC, Ackermann HH (2005). Comparison of continuous background infusion plus demand dose and demand-only parturient-controlled epidural analgesia (PCEA) using ropivacaine combined with sufentanil for labor and delivery. Int J Obstet Anesth.

[20] Lee AI, McCarthy RJ, Toledo P, Jones MJ, White N, Wong CA (2017). Epidural labor analgesia-fentanyl dose and breastfeeding success: a randomized clinical trial. Anesthesiology.

[21] Li Y, Hu C, Fan Y, Wang H, Xu H (2015). Epidural analgesia with amide local anesthetics, bupivacaine, and ropivacaine in combination with fentanyl for labor pain relief: a meta-analysis. Med Sci Monit.

[22] Ortner CM, Posch M, Roessler B, Faybik P, Rutzler K, Grabovica J, Kimberger O, Gustorff B (2010). On the ropivacaine-reducing effect of low-dose sufentanil in intrathecal labor analgesia. Acta Anaesthesiol Scand.

[23] Zhang L, Shu R, Zhao Q, Li Y, Wang C, Wang H, Yu Y, Wang G (2017). Preoperative but not postoperative Flurbiprofen Axetil alleviates Remifentanil-induced Hyperalgesia after laparoscopic Gynecological surgery: a prospective, randomized, double-blinded, trial. Clin J Pain.

[24] Ngan Kee WD, Ng FF, Khaw KS, Tang SPY, Koo AGP (2017). Dose-response curves for Intrathecal bupivacaine, Levobupivacaine, and Ropivacaine given for labor analgesia in nulliparous women. Reg Anesth Pain Med.

[25] Nakamura G, Ganem EM, Rugolo LM, Castiglia YM (2009). Effects on mother and fetus of epidural and combined spinal-epidural techniques for labor analgesia. Rev Assoc Med Bras (1992).

